# Effects of a Post-Discharge Care Program for Surgery Patients with Brain Tumor

**DOI:** 10.3390/healthcare13172179

**Published:** 2025-09-01

**Authors:** Taeyeong Yang, Saekyae Shin, Youngseon Ahn, Sohyune Sok

**Affiliations:** 1 Department of Nursing, College of Nursing, Dongguk University-Gyeongju, Gyeongju 38066, Republic of Korea; ty727yang@naver.com; 2 Department of Nursing, Graduate School, Kyung Hee University, Seoul 02447, Republic of Korea; purin0327@naver.com (S.S.); n0324@shhosp.co.kr (Y.A.); 3 College of Nursing Science, Kyung Hee University, Seoul 02447, Republic of Korea

**Keywords:** brain tumor, post-discharge, symptom clusters, adaptation, quality of life

## Abstract

Background/Objectives: Post-discharge interventions addressing psychological, informational, and practical needs of brain tumor surgery patients are limited. This study aimed to develop and examine the effects of a post-discharge care program for patients with benign brain tumors who underwent surgery. Methods: A quasi-experimental study with a nonequivalent control group pretest–post-test non-synchronized design was employed. The post-discharge care program was developed using the ADDIE model and delivered as an 8-week, 8-session program to 65 discharged patients (Intervention: *n* = 33, Control: *n* = 32). Outcomes were measured using the Memorial Symptom Assessment Scale (MSAS) for symptom clusters, Post-Discharge Coping Difficulty Scale (PDCDS) for post-discharge adaptation, and Functional Assessment of Chronic Illness Therapy (FACIT) for quality of life. Results: Significant group × time interactions were found between intervention and control groups for symptom clusters (F = 74.878, *p* < 0.001), post-discharge adaptation (F = 144.687, *p* < 0.001), and all quality of life domains: physical (F = 38.996, *p* < 0.001), social/family (F = 50.865, *p* < 0.001), emotional (F = 39.110, *p* < 0.001), and functional (F = 38.917, *p* < 0.001). The intervention group showed clinically meaningful improvements across all outcomes. Conclusions: This study demonstrates that the post-discharge care program was effective in improving symptom clusters, post-discharge adaptation, and quality of life in patients with benign brain tumors who underwent surgery. The program can contribute to achieving better health outcomes for this population in clinical practice.

## 1. Introduction

A brain tumor is a central nervous system disease that refers to tumors that occur in various structures in the brain and surrounding areas [1]. Patients with brain tumors experience systemic symptoms, including pain, fatigue, depression, anxiety, and neurological symptoms, including headaches, epileptic seizures, paralysis, visual disturbances, and hydrocephalus depending on the tumor’s location and characteristics [2]. Recent studies have shown that patients with primary or metastatic brain tumors simultaneously experience various symptom clusters—such as fatigue, gastrointestinal symptoms, emotional disturbances, and neurocognitive impairments—which negatively affect their quality of life, performance status, and functional ability [3]. Therefore, improving symptom clusters is crucial in symptom management during the recovery from brain tumor surgery [2]. Additionally, most healthcare systems have transitioned to models that favor early discharge to home or the community [4]. Consequently, there is a lack of social resources to address the psychological, informational, and practical needs of brain tumor surgery patients after discharge [1]. Hestevik et al. [4] reported that patients experience anxiety and safety concerns during the transition from hospital to home and may even feel at risk. Zhang et al. [2] emphasized that lack of information on continuous self-care after discharge may negatively impact patients’ adaptation in these situations. Therefore, it is necessary to provide information and enhance patients’ capacity to perform effective self-management after discharge [5]. The quality of life in patients with brain tumors has been shown to vary not only according to treatment-related factors but also in relation to financial hardship, time since diagnosis, and whether the patient is undergoing treatment or experiencing a recurrence [6]. Lee et al. [7] analyzed the quality of life of 96 patients who underwent brain tumor surgery and found that patients who had no neurological disorders after surgery, no experience of reoperation, and no complications reported high quality of life. However, patients who underwent brain tumor surgery may have more complex problems after discharge than patients with general cancer, which may lead to readmission in serious cases [8].

In the United States, the Affordable Care Act was implemented in 2012 to cut reimbursement for hospitals with high readmission rates [9]. Afterward, several plans were explored to help patients adjust to daily life after discharge and extend treatment at home. Robertson et al. [8] argued that the readmission rate within 30 days was reduced when nurses provided education on postoperative activities, medication, wound care, nutrition, and signs requiring medical treatment via phone 48 h after discharge. These results suggest that low-cost, small-scale post-discharge interventions can provide ongoing care plans, improve clinical outcomes, enhance patient engagement and satisfaction, and identify complications early to manage symptoms and reduce costly readmissions [10]. Recent studies have examined the course of recovery and functional outcomes following inpatient rehabilitation [11], as well as the impact of a brain tumor diagnosis on patients’ lives and their coping strategies during the surgical process [12]. However, most prior research has primarily focused on identifying factors influencing recovery, coping, and quality of life after discharge, with a notable lack of studies that develop and evaluate structured intervention programs to improve these outcomes. Furthermore, existing intervention studies tend to address single aspects—such as cognitive rehabilitation or physical therapy—without providing an integrated, multidisciplinary approach that combines physical, psychological, and social support. In particular, for patients undergoing surgery for benign brain tumors, there is a critical gap in post-discharge programs designed to manage symptom clusters, facilitate adaptation to daily life, and enhance overall quality of life [2,8]. To address this gap, the present study developed and evaluated an eight-week, structured, multidisciplinary post-discharge care program tailored to the unique needs of patients who underwent surgery for benign brain tumors. This program aims to provide foundational clinical evidence to support patients’ physical and psychological recovery, rehabilitation, and stable return to daily life.

**Figure 1:**
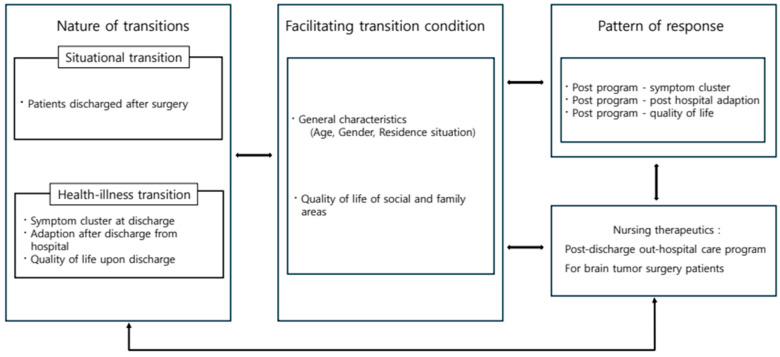
Conceptual framework of this study.

In developing the post-discharge care program, the components of Meleis’ transition theory [13] directly informed the program structure and content (Figure 1). First, the type of transition addressed included both situational transition—patients moving from the hospital to home after surgery—and health–illness transition, in which patients progress from a diseased state to recovery and adaptation. These transition types guided the selection of program goals, emphasizing smooth role adaptation and the restoration of daily functioning. Second, facilitating and inhibiting conditions identified in the theory—such as age, gender, living arrangements, family support, and baseline quality of life—were reflected in tailoring educational content and delivery methods. For example, patients with limited digital literacy received additional one-on-one technical guidance before online sessions. Third, response patterns, including changes in symptom clusters, post-discharge adaptation, and quality of life, were selected as primary outcome variables to evaluate the effectiveness of the intervention, consistent with Meleis’ emphasis on indicators of healthy transition. Finally, nursing interventions, as conceptualized in the theory, were operationalized through the eight structured weekly sessions of the program. Each session corresponded to specific needs during the transition process—for instance, early sessions (Sessions 1–3) addressed awareness of disease characteristics and complication prevention, middle sessions (Sessions 4–6) focused on skill-building in medication, diet, physical activity, and stress management, and later sessions (Sessions 7–8) supported social reintegration and redefinition of life goals. These sequential components were designed to facilitate progression through the transition process toward optimal adaptation and well-being.

This study aims to develop a post-discharge care program for patients undergoing brain tumor surgery and examine its effects on patients’ symptom clusters, post-discharge adaptation, and quality of life.

### Hypothesis

- There will be differences in symptom cluster scores between subjects who received the post-discharge care program and those who did not.
- There will be differences in post-discharge adaptation scores between subjects who received the post-discharge care program and those who did not.
- There will be differences in quality of life scores between subjects who received the post-discharge care program and those who did not.

## 2. Materials and Methods

### 2.1. Study Design and Participants

**Figure 2:**
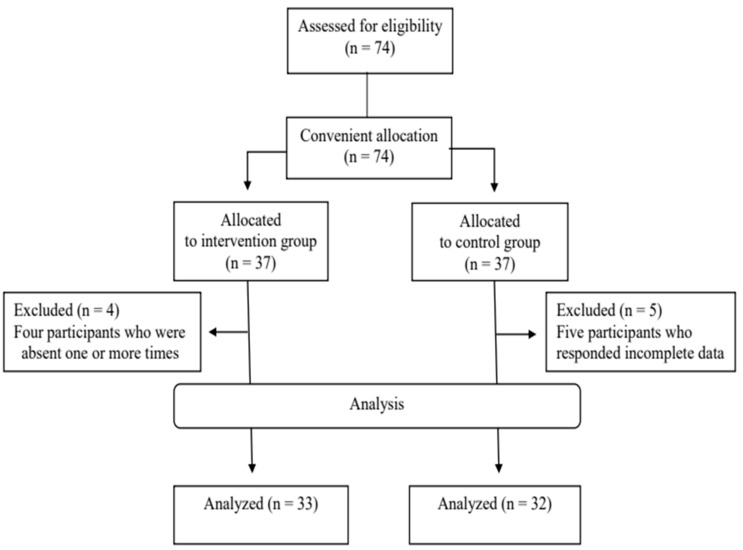
Flow diagram of subject progress.

This study was designed as a quasi-experimental study with a nonequivalent control group pretest–post-test non-synchronized design. The study subjects were 65 patients with benign brain tumors who visited the department of neurosurgery at K General Hospital in Seoul, underwent surgery, and were discharged from September, 2023 to February, 2024. Only 74 patients were screened and all 74 patients were assigned to the experimental or control group. The study participants were conveniently allocated to experimental and control groups without random assignment. During the research, four subjects in the intervention group and 5 subjects in the control group dropped out, and the research was ultimately conducted with 33 subjects in the intervention group and 32 subjects in the control group (Figure 2). The selection criteria were as follows: (1) patients aged 19 years or older who visited the department of neurosurgery at K General Hospital and underwent surgery for a benign brain tumor; (2) subjects who can communicate independently and read and write in Korean, and who can understand the program content and pre- and post-surveys and actively participate; (3) subjects who understood the study’s purpose and voluntarily agreed to participate in the study; and (4) eligible subjects for online ZOOM-based educational programs (those who possess a digital device such as a smartphone, tablet, or laptop, and can access ZOOM through themselves or a guardian). The exclusion criteria were as follows: (1) subjects diagnosed with cognitive impairment, such as dementia, or who have difficulty communicating or are unable to communicate independently and read Korean for other reasons; (2) subjects who participated in a similar intervention program within the past six months; and (3) subjects who were prescribed medications that could affect daily activities including sleeping pills. The sample size for each group was calculated as 31 by setting the significance level to 0.05, power (1-β) to 0.90, medium effect size (f) to 0.25, number of groups to 2, and number of repeated measurements to 2 in the G*power 3.1.9.2 program [14]. In the absence of similar prior studies, 74 subjects were recruited, 37 in each group considering the medium effect size (f = 0.25) and 20% dropout rate, as suggested by Cohen [15].

### 2.2. Development of a Post-Discharge Care Program

The post-discharge care program in this study was based on the Analysis, Design, Development, Implementation, Evaluation (ADDIE) model widely used as an instructional design (ID) model [16]. In the analysis stage, previous studies related to the research topic were analyzed. Some areas that were examined included disease characteristics and risk factors of brain tumors [17], post-operative adaptation [18], quality of life management [19], prevention and management of complications due to brain tumors [5,20], drug therapy [21], diet therapy [22], kinesitherapy [23], and hygiene management [24] for patients with brain tumors. In the design stage, the program’s session objectives and content were developed based on a comprehensive literature review and expert consultation. It was structured as an 8-week integrated management program designed to support postoperative adaptation in patients who had undergone brain tumor surgery. Sessions were conducted once weekly for 60 min each, with a standardized format consisting of an introduction (20 min), educational lecture and Q&A (30 min), and a wrap-up with assignment briefing (10 min). This multidisciplinary intervention addressed various domains, including physical recovery through exercise and dietary management; psychological support through stress-coping strategies and self-management techniques; and educational support through disease-related information and prognosis education. Each session incorporated tasks aimed at enhancing patients’ awareness and engagement in their own recovery process. The content covered a wide range of topics such as the characteristics and risk factors of brain tumors, treatment course and clinical progression, management of postoperative complications and emergencies, medication and dietary guidance, exercise strategies for recovery, stress management, rebuilding of social relationships, and reconstructing life after discharge. Detailed objectives and strategies for each session are presented in Table 1.

In the development stage, a draft in PPT format was created based on the learning objectives and detailed content composition. Afterward, feedback was received from a group of experts consisting of one nursing professor, three neurosurgeons, and two neurosurgery nurses to verify the composition, format, and content of the educational materials. The experts suggested that medical terms should be excluded as much as possible, the lecture content should be structured briefly, and the time to resolve patients’ real-life questions should be increased. To reflect the recommendations in the educational materials, most medical terms were excluded, and annotations were added where necessary to make them easier to understand. In addition, the slide presentations were reduced to about 10 slides per session, and the training time was organized to not exceed 30 min. A workbook in the form of a diary was also developed to help the subjects achieve their goals and continuously utilize them in their daily lives. The workbook was organized based on the educational topics selected for each session and included the following items: (1) recording physical, mental, and social conditions; (2) post-discharge adaptation and complications, and a week’s resolution to overcome them; (3) side effects of medications taken after discharge and a weekly diet and compendium of physical activities; (4) activities that the patient did for himself/herself for a week; (5) patient’s daily routine plan; (6) thinking about family and social relationships surrounding the patient; and (7) people and content of gratitude for today. It was designed to present and plan tasks for the week.

The intervention in this study was conducted solely by the researcher, a nurse specialist with over 15 years of clinical experience in neurosurgical care. The program began one week after discharge and was implemented once a week for a total of eight weeks, with each session lasting 60 min. To encourage participation, the same session was offered flexibly on either Monday or Wednesday, allowing participants to choose their preferred day. The first session, held during the patient’s first outpatient visit after discharge, was conducted face-to-face in a hospital meeting room. Sessions from Week 2 to Week 8 were delivered in real-time via the Zoom platform. For participants unfamiliar with the virtual platform, instructions were provided in advance together with their caregivers, and the researcher personally assisted with mobile device setup to improve accessibility. Each session consisted of three components: task review (20 min), education and Q&A (30 min), and summary with guidance for the next assignment (10 min). Session topics included understanding the disease, complication prevention, medication and dietary management, physical activity, self-management, restoring relationships, and restructuring life. Educational materials consisted of approximately 10 PowerPoint slides per session and a participant workbook in a diary format. The workbook included sections for recording physical, emotional, and social status, setting and reflecting on weekly goals, and writing a gratitude journal. To ensure consistency and fidelity of the program, the researcher used an educational checklist to verify whether the core components of each session were implemented. Attendance and completion of the workbook were also regularly monitored. Additionally, reminder text messages were sent the day before each session to promote engagement, and real-time phone support was provided to participants experiencing technical difficulties to facilitate smooth participation in the intervention.

During the evaluation stage, the differences in symptom clusters, post-discharge adaptation, and quality of life between the intervention and control groups were verified. The goals and detailed composition of the proposed program are shown in Table 1.

### 2.3. Measures

Study participant’s general characteristics survey consisted of 6 items in total, namely, gender, age, residential status, educational level, marital status, and duration of treatment with brain tumor.

To examine symptom cluster, the study used Portenoy et al.’s [25] Memorial Symptom Assessment Scale (MSAS) developed for general cancer patients, which was translated by Kim [26] to measure the symptom clusters of patients undergoing brain tumor surgery. MSAS measures 32 different symptom cluster items that cancer patients can experience, and patients self-evaluate the frequency, severity, physical and psychological pain and discomfort. The severity of symptoms is measured on a scale from 1 to 4 points, where a higher score indicates a higher severity. The total score of the 32 items ranges from a low score of 32 to a high score of 128, where a higher total score represents a more severe symptom cluster. At the time of development, Cronbach’s α was 0.88, and in Kim’s study [26], Cronbach’s α was 0.83. In this study, Cronbach’s α was 0.96.

To examine post-discharge adaptation, this study utilized Weiss and Piacentine’s [27] Post-Discharge Coping Difficulty Scale (PDCDS), which was translated by Kim [28] to fit the Korean situation, to measure post-discharge adaptation. This tool consists of 10 items, including overall stress in life perceived by patients, difficulties in the recovery process, difficulty in self-management, difficulty in managing medical conditions, difficulties experienced by family and friends, degree of need for emotional support, confidence in self-management, difficulty in managing medical needs, and degree of adaptation at home. Each item is measured on an 11-level scale from 0 to 10 points. The total score of the 10 items ranges from 0 to 100 points, where a higher total score means that the subject has more difficulty in adaptation after discharge. At the time of development, Cronbach’s α was 0.91, and in Kim’s study [28], Cronbach’s α was 0.88. In this study, Cronbach’s α was 0.95.

To examine quality of life, the research adopted Cella’s [29] Functional Assessment of Chronic Illness Therapy (FACIT), which was translated by Kim et al. [30] to measure the quality of life of patients undergoing brain tumor surgery. This tool covers 27 items that measure the quality of life in the physical, social family, emotional, and functional domains. Each item is measured on a five-level scale from 0 to 4 points. The total score of the 27 items ranges from 0 to 108 points, where a higher total score shows a higher quality of life. At the time of development, Cronbach’s α of the quality of life tool was 0.89, and in Kim et al.’s study [30], Cronbach’s α was 0.87. In this study, Cronbach’s α = 0.83 for the physical domain, 0.70 for the social and family domain, 0.83 for the emotional domain, and 0.74 for the functional domain. In this study, total Cronbach’s α was 0.81.

The measurement tools in this study were translated and back-translated twice by a native English-speaking nursing professor and a brain tumor specialist, respectively, to enhance the validity and reliability of the tools.

### 2.4. Data Collection

This study was conducted after receiving research approval from the Institutional Review Board of K General Hospital in Seoul. Considering the risk of experimental diffusion and the difficulty in recruiting two groups at the same time, a nonequivalent control group pretest–post-test non-synchronized design was used, recruiting the intervention group after all procedures in the control group were completed. The intervention group was recruited from patients whose discharge dates were set after surgery. The patients in the intervention group were examined during each session to ensure the smooth progress of the program. Before starting the week 1 education, a pre-survey was conducted for the intervention group. From week 2 to week 8, non-face-to-face education on the post-discharge care program was conducted via online conference once a week at 60 min per session, eight times for eight weeks, until the end of the intervention group’s schedule. For older adults or those unfamiliar with virtual platforms, caregivers assisted with accessing ZOOM and using the workbook during the initial session. After completing all programs, a post-survey was conducted. For the control group, a phone call was provided the day before the outpatient visit through a happy call, and a pre-test was conducted at the outpatient clinic one week after discharge. No other treatment was provided, except for two regular phone calls according to the next outpatient visit schedule, and a post-survey was conducted during the outpatient visit eight weeks later. To minimize contamination, the intervention group was recruited only after the control group had completed the full procedures. No educational or supportive intervention was provided to the control group. Apart from two routine follow-up phone calls (for outpatient scheduling and survey guidance), the control group received no additional contact from the research team. Notably, emergency department visits and readmissions were not included as outcome variables in this study, and no participants experienced such events during the 8-week study period.

### 2.5. Ethical Considerations

This study was conducted after receiving approval from the K General Hospital Institutional Review Board (KBSMC 2023-04-036). There was no separate trial registration number. Only subjects who gave written consent were allowed to participate in the study, and the consent form for research participation included information on the anonymity and confidentiality of the subjects. The subjects were asked to fully understand this information and voluntarily sign the consent form. In addition, it was clearly and easily explained that they could freely withdraw their participation at any time during the study and that there would be no disadvantages in withdrawing. The collected data were stored in a locked place in the researcher’s personal office and will be stored for the period set by the relevant ethics agency after the end of the study and then destroyed. All collected information was processed with code numbers and initials, and information about the subjects was not identifiable. Considering ethical issues, the program was applied to control group patients who wished to receive the program after the end of this study.

### 2.6. Statistical Analysis

The collected data were analyzed using the SPSS PC+ Version 23.0 program. Frequency analysis and descriptive statistics analysis were performed to analyze the general characteristics of the study participants. Independent t-test and chi-square test were performed to examine the prior homogeneity between the experimental and control groups. Two-way repeated measures ANOVA was performed to examine the effect of the post-discharge out-hospital care program.

## 3. Results

### 3.1. General Characteristics of the Study Participants and Homogeneity

Table 2 shows that when analyzing the gender, age, residential status, highest level of education, marital status, and duration of treatment with brain tumor of the intervention and control groups, there were no statistically significant differences between the two groups, confirming that they suggest similarities between the two groups (Table 2).

### 3.2. Effects of Post-Discharge Care Program

To verify the homogeneity of the main dependent variables (symptom clusters, post-discharge adaptation, and quality of life) between the two groups, a two-way repeated measures ANOVA used to analyze changes over time and the interaction effect between groups, performed statistical control at the multivariate level. There were no statistically significant differences in the main dependent variables between the two groups (Table 3).

**Figure 3:**
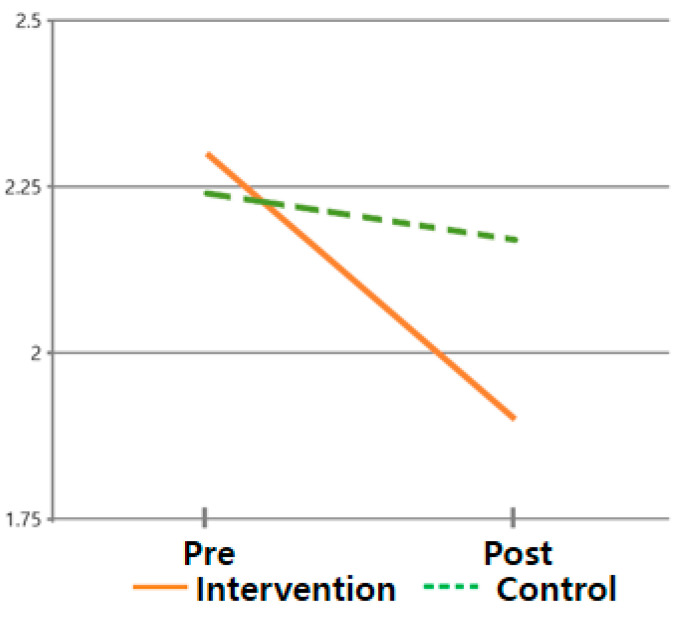
Changes in symptom clusters.

The hypothesis that ‘there will be differences in symptom cluster scores between subjects who received the post-discharge care program and those who did not’ was verified. For the symptom cluster, the pre-mean (standard deviation) of the intervention group was 2.30 (0.581), and the post mean (standard deviation) was 1.90 (0.630). The pre-mean (standard deviation) of the control group was 2.24 (0.379), and the post mean (standard deviation) was 2.17 (0.423). This means that the symptom cluster of the intervention group that applied the post-discharge care program improved, while the symptom cluster of the control group did not improve, supporting this hypothesis. Hence, the difference in the change in symptom cluster was statistically significant between times (F = 157.742, *p* < 0.001) and between groups × times (F = 74.878, *p* < 0.001) but not between groups (F = 0.641, *p* = 0.426) (Table 3 and Figure 3).

**Figure 4:**
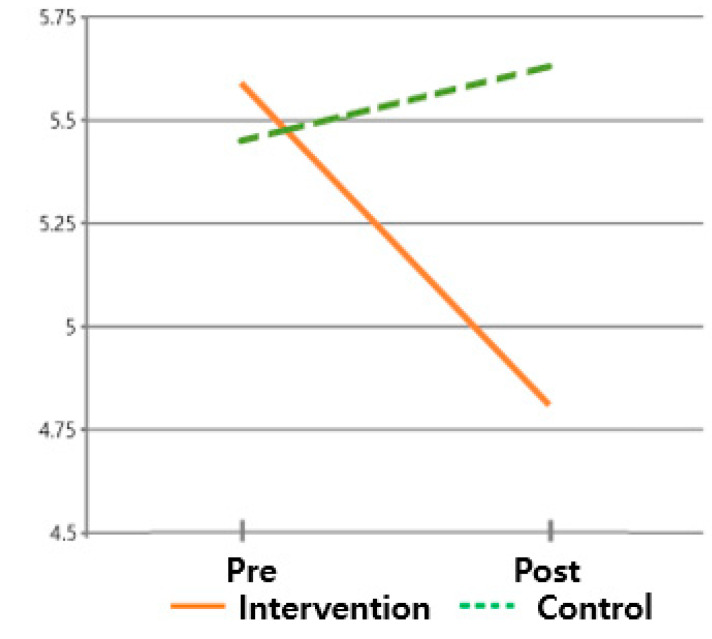
Changes in post-discharge adaptation.

The hypothesis that ‘there will be differences in post-discharge adaptation scores between subjects who received the post-discharge care program and those who did not’ was validated. For the post-discharge adaptation, the pre-mean (standard deviation) of the intervention group was 5.59 (1.541), and the post mean (standard deviation) was 4.81 (1.845). On the other hand, the pre-mean (standard deviation) of the control group was 5.45 (1.514), and the post mean (standard deviation) was 5.63 (1.453). The post-discharge adaptation of the intervention group enhanced, while the post-discharge adaptation level of the control group did not improve, supporting this hypothesis. These findings suggest that the difference in the post-discharge adaptation change was statistically significant between times (F = 56.691, *p* < 0.001) and between groups × times (F = 144.687, *p* < 0.001) but not between groups (F = 0.760, *p* = 0.387) (Table 3 and Figure 4).

**Figure 5:**
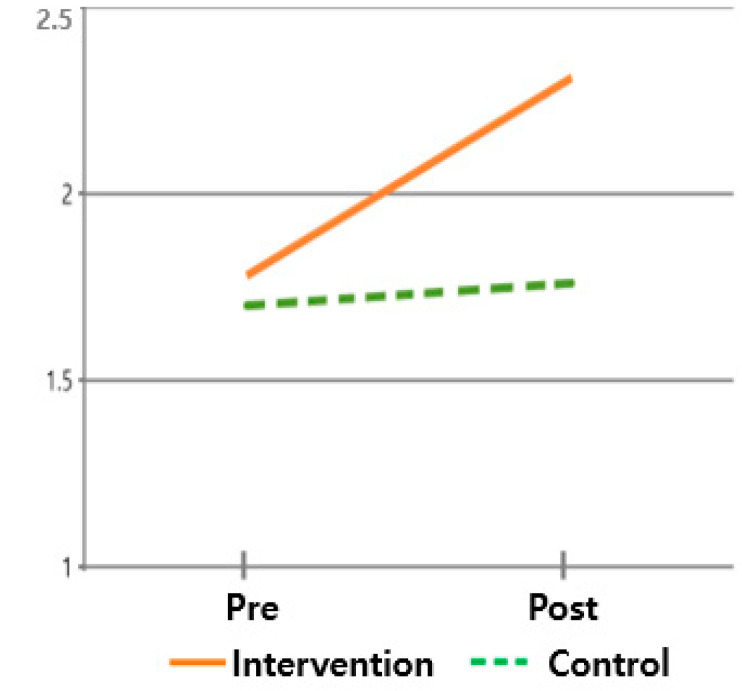
Changes in QOL of physical area.

**Figure 6:**
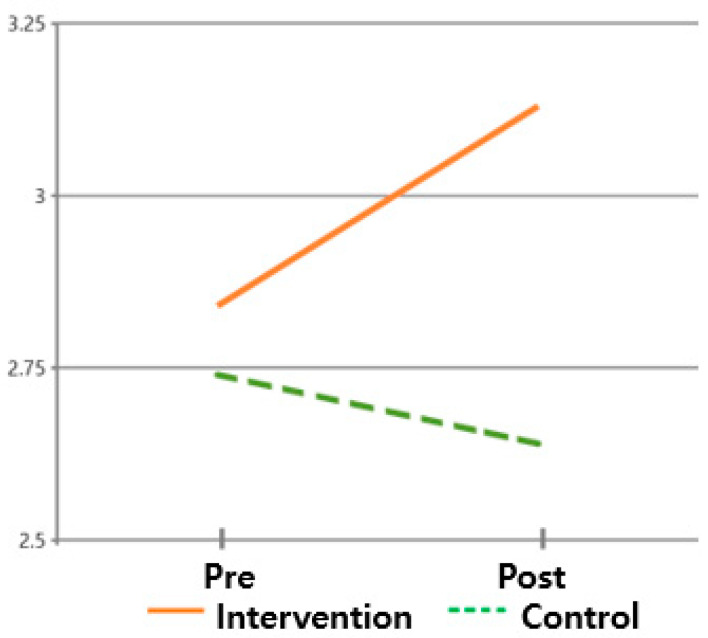
Changes in QOL of social and family areas.

**Figure 7:**
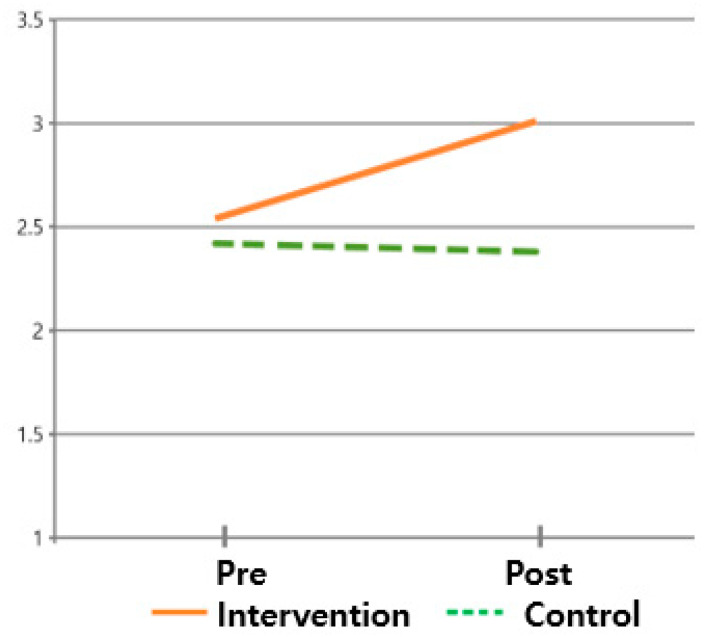
Changes in QOL of emotional area.

**Figure 8:**
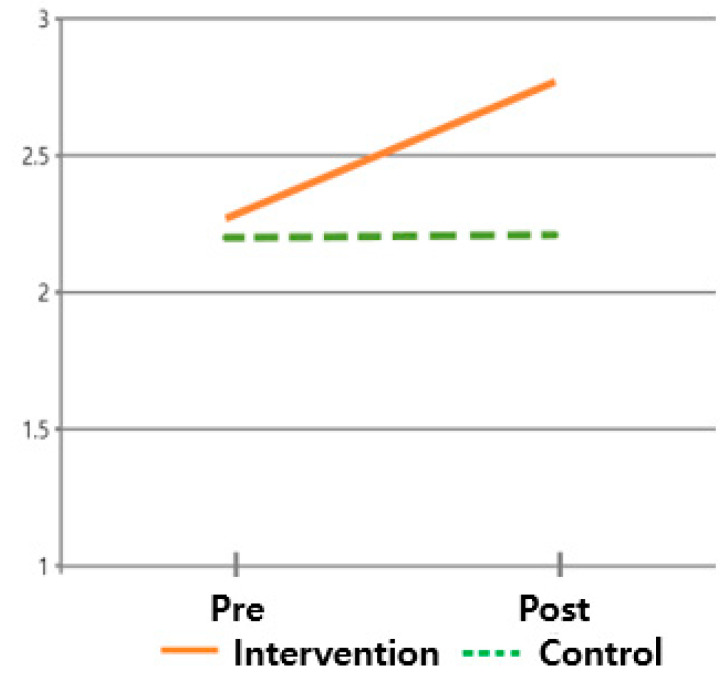
Changes in QOL of functional area.

The hypothesis that ‘there will be differences in quality of life scores between subjects who received the post-discharge care program and those who did not’ was verified. The differences in the four domains (i.e., physical, social/family, emotional, and functional) of quality of life between the intervention and control groups were all significant. Additionally, the main effect of the difference between times (F = 62.743, *p* < 0.001) and the interaction of the difference between groups and times (F = 38.996, *p* < 0.001) in the physical domain was significant, while the main effect of the difference between groups (F = 3.372, *p* = 0.071) was insignificant. However, in the remaining three domains, the main effect of the difference between times, the main effect of the difference between groups, and the interaction of the difference between groups and times were all significant, supporting this hypothesis (Table 3, Figure 5, Figure 6, Figure 7 and Figure 8).

## 4. Discussion

This study developed and applied a post-discharge care program for patients who underwent brain tumor surgery and then examined the effectiveness of the program. The intervention group that received the post-discharge care program had significantly lower pre- and post-scores of the symptom cluster than the control group, reflecting that the post-discharge care program improved various symptoms, including physical and psychological pain, of the subjects. Zhao [31] reported that a reminiscence therapy-based nursing program, delivered twice a month over a 12-month period to 150 postoperative brain tumor patients, significantly reduced levels of anxiety and depression while improving patient satisfaction. Similarly, O’Donovan et al. [32], through a systematic scoping review, found that various rehabilitation interventions contributed to improvements in both cognitive and physical functions, ultimately enhancing the quality of life for patients with brain tumors. These results support the present study’s proposition that the education and intervention program provided to patients with brain tumors reduces anxiety and depression symptoms psychologically, relieves stress physically, and helps maintain physical ability, thereby alleviating physical and psychological symptoms. However, it is difficult to comprehensively evaluate physical and psychological functions after discharge as previous studies focused only on specific functions such as cognitive rehabilitation and motor rehabilitation. Richard et al. [33] claimed that patients’ behavioral and cognitive functions were enhanced after participating in a goal management training program that combined physical and psychological training eight times for eight weeks in a randomized experiment targeting 25 patients with brain tumors. This is similar to the period and composition of the current research. Comprehensively, the study’s proposed program aimed to help prevent physical and psychological symptoms and complications that may occur after brain tumor surgery by providing education and practice related to the characteristics and risk factors of the disease, management of complications due to surgery, drug therapy, and kinesitherapy. Thus, it is expected that symptoms can be significantly improved. Next, participants in the intervention group who received the post-discharge care program demonstrated significant improvements across four domains of quality physical, social/family, emotional, and functional. In general, the health-related quality of life (HRQoL) of patients with brain tumors is known to decline due to the burden of multiple physical symptoms and psychological distress, with pain and depression being particularly influential factors [34]. Pain is frequently cited as one of the most critical determinants of HRQoL, and its co-occurrence with depressive symptoms is strongly associated with further deterioration in overall well-being. Additionally, HRQoL is closely linked to patients’ functional status [35], with greater physical limitations—such as fatigue, reduced ability to perform daily activities, and impaired motor function—contributing to lower quality of life scores [36]. These physical and psychological factors rarely occur in isolation; rather, they often present simultaneously, interacting in ways that compound their negative impact on patients’ well-being. Therefore, managing even a single symptom effectively may produce positive spillover effects across other domains. The comprehensive approach of our post-discharge care program, which addressed both physical symptoms and psychological challenges, appears to have contributed meaningfully to the overall enhancement of participants’ quality of life. These findings are consistent with those reported by Ownsworth et al. [37], who evaluated the “Making Sense of Brain Tumour” program among 82 patients and found that the intervention group reported significantly reduced depressive symptoms and improved quality of life and anxiety levels. Similarly, a recent systematic review by O’Doherty et al. [38] examining interventions for brain tumor patients—including physical rehabilitation, cognitive therapy, and psychosocial support—found that approximately 90% of interventions resulted in significant improvements in at least one indicator of patient well-being. Their meta-analysis further confirmed that quality of life scores was significantly higher in intervention groups compared to controls. In particular, home-based psychosocial programs and standardized rehabilitation interventions have been shown to be effective in enhancing HRQoL among this population. Given these findings, there is a clear need for ongoing development and rigorous evaluation of integrative strategies that target both physical and psychological symptoms in brain tumor patients. The post-discharge care program implemented in this study was grounded in such a comprehensive framework and demonstrated significant improvements not only in participants’ quality of life but also in their perceived level of post-discharge adjustment. Following a brain tumor diagnosis, patients and their families often undergo profound life changes. From the initial shock of diagnosis to eventual acceptance, they face a range of emotional responses as they try to understand and adapt to their new reality [39]. As the illness progresses, family roles and interpersonal dynamics may shift and require renegotiation. However, in many cases, treatment begins urgently following diagnosis, often leaving patients and families with insufficient time to process the diagnosis or prepare for the subsequent care trajectory, including post-discharge planning [39]. This makes the early post-diagnosis period a critical window for intervention. Structured and integrative post-discharge care during this phase may serve not only to improve patients’ quality of life but also to support their adaptive capacity as they navigate the complex challenges of living with a brain tumor. Additionally, patients’ treatment needs and timely education and intervention will play a vital role in improving patients’ health, function, and post-discharge adaptation, as implied in the results of this study [2]. In this context, the post-discharge care program developed in this study recruited subjects who had the same disease and surgery after discharge. Before the program implementation, the participants formed a bond of empathy by connecting as colleagues and social supporters. It is considered that the analysis of family relationships and social relationships may have affected the psychological aspect.

As for implications, the proposed post-discharge care program for patients undergoing brain tumor surgery has been effective in this study. Using it in the community can help support more patients with brain tumors in terms of comprehensive health management, post-discharge adaptation to daily life, and improvement of quality of life. The research findings suggest that concrete effective guidelines are required to comprehensively manage the subjects’ symptoms, post-discharge adaptation, and quality of life for patients who have undergone brain tumor surgery to recover and successfully return to their daily lives. Above all, administrative and institutional supplementation will be necessary for sustainable programs. Further research needs to validate the effectiveness of this program by applying and implementing it to a larger number of patients who underwent brain tumor surgery. The program’s effectiveness also needs to be confirmed using a tool that can specifically identify symptom clusters in patients who underwent brain tumor surgery. This study was conducted solely by the researcher, a neurosurgical nurse with over 15 years of clinical experience. However, future research needs to design programs that include formal caregiver education to promote post-discharge self-care and improve quality of life. Given the crucial role caregivers play in discharge planning, interventions should be developed to incorporate a more comprehensive multidisciplinary approach and a family-centered educational system. Also, future research needs to improve the validity and practicality of the program by including qualitative and quantitative evaluations of participant satisfaction, perceived burden of the intervention, and program usability.

In study limitations, this quasi-experimental study only targeted a small number of research participants who visited a single medical institution, underwent surgery, and were discharged. Therefore, there are limitations in generalizing the results to all patients who underwent brain tumor surgery. Also, some subjects dropped out due to lack of participation as the number of training sessions was large, and there were time constraints for one tutor to handle the task. The tool used in this study to measure symptom clusters was developed to evaluate all types of cancer patients and has limitations in classifying and evaluating specific symptoms that appear in patients who underwent brain tumor surgery. Although all participants were diagnosed with benign brain tumors based on medical records, the study did not collect detailed tumor characteristics such as histology, grade, location, or surgical complexity. Given the clinical heterogeneity, even among benign tumors, the absence of these variables is a notable limitation. This limitation stems from the single-institution nature of the study and the restricted sample size. Future research should incorporate these tumor-related variables and adopt multivariate regression or matching methods to minimize confounding effects and improve the generalizability of the findings. In addition, this study did not assess participants’ functional status using standardized outcome measures such as the Karnofsky Performance Status, Barthel Index, or modified Rankin Scale. Instead, clinical indicators such as mobility and home discharge were briefly described. This limitation hinders objective analysis of functional recovery. Future studies should incorporate validated functional assessment tools to provide more reliable and quantitative evaluations. This study focused on evaluating short-term outcomes within 30 days after discharge. However, the long-term effects of the post-discharge care program, including 90-day readmission rates or sustained health outcomes, were not assessed. Future research should consider extended follow-up to determine the durability of intervention effects. This study utilized an online intervention program delivered via ZOOM, which may have introduced participation barriers related to internet access and digital literacy. Although no standardized screening tool was used to assess digital competency, participants were required to have basic proficiency with smart devices to be included. In some cases, especially among older adults or those unfamiliar with virtual platforms, caregivers assisted during the initial sessions to help with ZOOM access and workbook use. These differences in digital accessibility may have influenced participation rates and intervention effectiveness, posing a potential limitation to the generalizability of the findings. Future studies should consider incorporating alternative delivery methods to better accommodate individuals with limited digital access or literacy. This study employed a quasi-experimental design without randomization or allocation concealment. As such, there is a potential risk of selection bias, which may have influenced the comparability between groups at baseline and the internal validity of the findings. Although baseline homogeneity was verified through statistical testing, the absence of random assignment limits the ability to establish causality. Future research should consider randomized controlled trials with proper allocation concealment to strengthen the evidence for the program’s effectiveness.

## 5. Conclusions

In conclusion, the post-discharge care program developed in this study was effective in improving symptom clusters, post-discharge adaptation, and quality of life of patients who underwent brain tumor surgery. Based on this, we suggest that a post-discharge care program that can support comprehensive mental and physical health management, self-management, and qualitative transition to a healthy and self-directed life for patients who underwent brain tumor surgery after discharge should be provided and utilized.

This study is significant in that it organized and offered an integrated program that can affect surgical patients’ physical conditions (e.g., cognition, function, and language) and psychological conditions. This program is systematically managed by nurses from the hospital to post-discharge, considering the reality that it is difficult to receive proper discharge education and post-discharge care due to the recent shortened hospitalization period. The post-discharge care program developed in this study utilized an online platform so that patients with brain tumors who have limited accessibility could easily participate in their daily lives.

## Figures and Tables

**Table 1:** Post-discharge care program for surgery patients with brain tumor.

Session	Topic/Objectives by Session	Activities
1	Introduction to participants Introduction to the program Promote understanding and empathy for the meaning and goals of the program. Understand the disease characteristics and risk factors of brain tumors and accurately recognize the patient’s current physical, mental, and social status.	Early (20 min): Explain the program structure, goals, and progress, and promote self-introduction and friendship for each participant
		Middle (30 min): Teach ‘disease characteristics and risk factors of brain tumors’ and conduct Q&A.
		Late (10 min): Conduct a pre-survey on symptom cluster improvement, post-discharge adaptation, and quality of life to verify the program’s effectiveness
2	Understand the treatment patterns and progress of brain tumors. Understand precautions after discharge. Recognize and report changes in the mind and body due to surgery, personal and social difficulties, etc. Understand and explain the goals, needs, and expectations of mental and physical health, post-discharge adaptation, and quality of life management.	Early (20 min): Report and share assignment performance.
		Middle (30 min): Discuss ‘treatment and progress of brain tumors’ and have Q&A.
		Late (10 min): Summarize the session’s contents and suggest the next task.
3	Prevent complications and sequelae. Understand emergencies, emergency transfer situations, and prevention and coping methods after discharge. Understand the causes, prevention, and management of complications and sequelae. Recognize and manage brain tumor-specific complications such as cognitive fatigue, seizures, and neurological impairments	Early (20 min): Report and share assignment performance.
		Middle (30 min): Teach ‘brain tumor-related complications, sequelae, and emergencies’ and have Q&A.
		Late (10 min): Summarize the session’s contents and suggest the next task.
4	Understand drug therapy and diet therapy for comprehensive improvement of physical and mental health. Understand the efficacy, administration methods, side effects, etc., of drugs related to brain tumors. Explain the importance of proper dietary management and precautions during the recovery process.	Early (20 min): Report and share assignment performance
		Middle (30 min): Teach ‘drug therapy and diet therapy for patients undergoing brain tumor surgery’ and have Q&A.
		Late (10 min): Summarize the session’s contents and suggest the next task.
5	Understand the effects and importance of recovery and rehabilitation through kinesitherapy. Practice kinesitherapy to improve physical and mental health.	Early (20 min): Report and share assignment performance.
		Middle (30 min): Teach ‘kinesitherapy for patients undergoing brain tumor surgery’ and have Q&A.
		Late (10 min): Summarize the session’s contents and suggest the next task.
6	Self-management, lifestyle, and hygiene management for post-discharge adaptation and improvement of quality of life Cultivate bathing and hygiene management, regular lifestyle habits, and understand stress management methods. Explore and understand the patient’s current condition, identity, and meaning of existence, and embrace them as they are. Understand mind control and stress management know-how that turns the pain caused by illness into an opportunity for human maturity.	Early (20 min): Report and share assignment performance.
		Middle (30 min): Explain ‘post-discharge adaptation, meaning of quality of life’ and have Q&A.
		Late (10 min): Summarize the session’s contents and suggest the next task.
7	Reflect on and reestablish social relationships for post-discharge adaptation and quality of life improvement. Analyze the family and social relationships surrounding the patient and reflect on the problems with an open mind. Use the patient’s strengths to form positive values and seek ways to expand them socially.	Early (20 min): Report and share assignment performance.
		Middle (30 min): Teach ‘post-discharge adaptation, harmonious social relationships’ and have Q&A.
		Late (10 min): Summarize the session’s contents and suggest the next task.
8	Redefine the meaning of life. Understand the new meaning of life after discharge, the value of true happiness in life, and embrace life as it is. Summary and reflection on the program	Early (20 min): Report and share assignment performance.
		Middle (30 min): Discuss the post-discharge program comprehensively and allot time for Q&A.
		Late (10 min): Conduct a pre-survey on symptom cluster improvement, post-discharge adaptation, and quality of life to verify the program’s effectiveness.

**Table 2:** General characteristics of the study participants and homogeneity.

Characteristic	Intervention Groups (*n* = 33) *n* (%)	Control Group (*n* = 32) *n* (%)	X^2^	*p* Value
Gender			0.799	0.455
Male	17 (51.5)	20 (62.5)		
Female	16 (48.5)	12 (37.5)		
Age (years)			0.207	0.837
30–39	2 (6.1)	2 (6.3)		
40–49	5 (15.2)	4 (12.5)		
50–59	8 (24.2)	10 (31.3)		
60–69	13 (39.4)	11 (34.4)		
70 and over	5 (15.2)	5 (15.6)		
Residential status			7.719	0.256
Alone	9 (27.3)	5 (15.6)		
With a spouse	4 (12.1)	1 (3.1)		
With one or more direct descendants	5 (15.2)	2 (6.3)		
With two or more immediate family members (a spouse, children, etc.)	14 (42.4)	19 (59.4)		
With relatives	-	2 (6.3)		
With non-blood acquaintances	1 (3)	-		
Other	-	3 (9.4)		
Educational level			6.196	0.196
High school graduate	11 (33.3)	4 (12.5)		
Junior college graduate	4 (12.1)	9 (28.1)		
4-years university graduate	14 (42.4)	12 (37.5)		
Graduate school or higher	2 (6.1)	3 (9.4)		
Other	2 (6.1)	4 (12.5)		
Marital status			2.052	0.436
Married	18 (54.5)	22 (68.8)		
Not married	5 (15.2)	5 (15.6)		
Single household	10 (30.3)	5 (15.6)		
Duration of treatment with brain tumor (month)			2.318	0.591
Less than 3	27 (81.8)	27 (84.4)		
3–6	1 (3)	3 (9.4)		
6–9	2 (6.1)	1 (3.1)		
9–12	3 (9.1)	1 (3.1)		

**Table 3:** Effects of post-discharge care program (*n* = 65).

Variables	Group	Time			Sources	
		Pre Mean (SD)	Post Mean (SD)		F	*p* Value
Symptom clusters	Inter.	2.30 (0.581)	1.90 (0.630)	Group Time Group*Time	0.641 157.742 74.878	0.426 <0.001 * <0.001 *
	Cont.	2.24 (0.379)	2.17 (0.423)			
Post-discharge adaptation	Inter.	5.59 (1.541)	4.81 (1.845)	Group Time Group*Time	0.760 56.691 144.687	0.387 <0.001 * <0.001 *
	Cont.	5.45 (1.514)	5.63 (1.453)			
QoL^†^ of physical area	Inter.	1.78 (0.777)	2.31 (0.884)	Group Time Group*Time	3.372 62.743 38.996	0.071 <0.001 * <0.001 *
	Cont.	1.70 (0.592)	1.76 (0.512)			
QoL^†^ of social and family areas	Inter.	2.84 (0.411)	3.13 (0.538)	Group Time Group*Time	9.575 11.013 50.865	0.003 * 0.002 * <0.001 *
	Cont.	2.74 (0.309)	2.64 (0.283)			
QoL^†^ of emotional area	Inter.	2.54 (0.546)	3.01 (0.729)	Group Time Group*Time	5.840 27.295 39.110	0.019 * <0.001 * <0.001 *
	Cont.	2.42 (0.648)	2.38 (0.629)			
QoL^†^ of functional area	Inter.	2.27 (0.516)	2.77 (0.652)	Group Time Group*Time	5.123 40.326 38.917	0.027 * <0.001 * <0.001 *
	Cont.	2.20 (0.585)	2.21 (0.583)			

Inter.: Intervention group; Cont.: Control group; Pre: Pre-intervention; Post: Post-intervention; Mean: estimated Mean; SD: Standardized Deviation; QoL^†^: Quality of life; * *p* < 0.05.

## Data Availability

The data that support the findings of this study are available from the corresponding author upon reasonable request.
